# Inheritance and Quantitative Trait Locus Mapping of *Fusarium* Wilt Resistance in Cucumber

**DOI:** 10.3389/fpls.2019.01425

**Published:** 2019-12-02

**Authors:** Jingping Dong, Jun Xu, Xuewen Xu, Qiang Xu, Xuehao Chen

**Affiliations:** School of Horticulture and Plant Protection, Yangzhou University, Yangzhou, China

**Keywords:** cucumber, *Fusarium* wilt, inheritance, quantitative trait locus fine mapping, resistance genes

## Abstract

*Fusarium* wilt (FW) is a very serious soil-borne disease worldwide, which usually results in huge yield losses in cucumber production. However, the inheritance and molecular mechanism of the response to FW are still unknown in cucumber (*Cucumis sativus* L.). In this study, two inbred cucumber lines Superina (P_1_) and Rijiecheng (P_2_) were used as the sensitive and resistant lines, respectively. A mixed major gene plus polygene inheritance model was used to analyze the resistance to FW in different generations of cucumber, namely, P_1_, P_2_, F_1_ (P_1_×P_2_), B_1_, and B_2_, obtained by backcrossing F_1_ plants with Superina (B_1_) or Rijiecheng (B_2_), and F_2_, obtained by self-crossing the F_1_ plants. After screening 18 genetic models, we chose the E-1 model, which included two pairs of additive-dominance-epistatic major genes and additive-dominance polygenes, as the optimal model for resistance to FW on the basis of fitness tests. The major effect quantitative trait locus (QTL) *fw2.1* was detected in a 1.91-Mb-long region of chromosome 2 by bulked-segregant analysis. We used five insertion/deletion markers to fine-map the *fw2.1* to a 0.60 Mb interval from 1,248,093 to 1,817,308 bp on chromosome 2 that contained 80 candidate genes. We also used the transcriptome data of Rijiecheng inoculated with *Fusarium oxysporum* f. sp. *cucumerinum* (Foc) to screen the candidate genes. Twelve differentially expressed genes were detected in *fw2.1*, and five of them were significantly induced by FW. The expression levels of the five genes were higher in FW-resistant Rijiecheng inoculated with Foc than in the control inoculated with water. Our results will contribute to a better understanding of the genetic basis of FW resistance in cucumber, which may help in breeding FW-resistant cucumber lines in the future.

## Introduction

Cucumber (*Cucumis sativu*s L.) is an economically important vegetable crop that ranks fourth in vegetable production worldwide. In 2017, the cultivated area in China was 1.24 million hectares with an annual output of 64.88 million tons, respectively accounting for 54.4% and 77.5% of the world total 2017 (http://faostat3.fao.org/). In the cucumber production season, *Fusarium* wilt (FW), downy mildew, and powdery mildew are the three main diseases found in China (Cao et al., 2007). FW of cucumber occurs more readily and seriously under a continuous cropping system, with incidences ranging from 30% to 90%, leading to huge loss of cucumber output (Pu et al., 2011; Zhou and Wu, 2012).

FW caused by *Fusarium oxysporum* f. sp. *cucumerinum* Owen (Owen, 1955), which is a forma specialis that infects the vascular bundles of cucumber, leads to necrotic lesions on the stem base, foliar wilting and eventually whole plant wilt, and even death (Zhou et al., 2008; Ye et al., 2004). Until now, there are no methods to effectively control the occurrence and harm of cucumber FW (Ye et al., 2004; Yuan et al., 2003).

Understanding the inheritance of FW resistance is an important step in developing resistant breeding resources and in breeding resistant varieties. The inheritance of cucumber resistance to FW has been studied for a long time, but the results are inconsistent (Pierce and Wehner, 1990; Vakalounakis, 1993). Vakalounakis, (2015) found that cucumber resistance to FW was controlled by a single gene, whereas others have suggested that cucumber FW resistance was regulated by multiple genes (Liu et al., 2003; Wang, 2005). To date, the inheritance of cucumber FW resistance remains poorly understood. Disease-resistant traits are quantitative traits that are controlled by multiple genes, which are generally located in multiple effect quantitative trait loci (QTLs; Hiroki et al., 2013; Falconer and Mackay, 1996). Bulked-segregant analysis (BSA) is an effective method for identifying DNA markers tightly linked to FW resistance (Giovannoni et al., 1991; Michelmore et al., 1991). For example, Li et al. (2018) mapped powdery mildew resistance genes to a region of chromosome 1A of wheat, and Zhang et al. (2018) confirmed that resistance to downy mildew and powdery mildew shared common candidate intervals on chromosome 5 of cucumber (Zhang et al., 2018) by BSA. DNA samples from soybean with opposite phenotypes were subjected to BSA to detect DNA markers that exhibited differences between the two different samples to identify the QTL (Mansur et al., 1993). Genome-wide insertion/deletion (InDel) markers have been used for fine mapping of important economical traits in rice (Feng et al., 2005; Li et al., 2017), wheat (Chen et al., 2005; Shang, 2009), and tomato (Su et al., 2019). In this study, a mixed major gene plus polygene inheritance model was used to analyze FW resistance in cucumber, and the major effect QTL of FW was investigated by BSA. We combined transcriptome data and mapping data to detect the key candidate resistance genes in cucumber after inoculation with Foc.

## Materials and Methods

### Cucumber Materials and Treatments

The two cucumber inbred lines, Superina and Rijiecheng, that showed susceptibility and resistance to FW, respectively, were used in this study. Superina (female parent, P_1_) and Rijiecheng (male parent, P_2_) were used to construct different generations, namely, F_1_, B_1_ (F_1_×Superina), B_2_ (F_1_×Rijiecheng), and F_2_. The F_1_ generation (P_1_×P_2_) was planted in Autumn 2016 in a greenhouse at the experimental farm of the Department of Horticulture in Yangzhou University, and the B_1_, B_2_, and F_2_ generations were obtained by backcrossing the F_1_ plants with Superina (B_1_) or Rijiecheng (B_2_) and self-crossing the F_1_ plants (F_2_) in Spring 2017. The 200 P_1_, 200 P_2_, 200 F_1_, 200 B_1_, 200 B_2_, and 1500 F_2_ plants were planted in 36-cavity plates filled with aseptic organic substrates (N, P, and K = 40–60 g/kg, humus ≥350 g/kg, pH = 6.5–7.5) in the greenhouse in Autumn 2017. Another 500 F_2_ and 200 F_2_ were planted in Autumn 2018 and Spring 2019, respectively. Seedlings at the second true leaf stage were inoculated with the Foc suspension (concentration 10^6^ conidia/ml) using the root irrigate method. The disease classification scale was as follows: grade 0, asymptomatic; grade 1, slightly discolored stem base and cotyledon; grade 2, necrotic patches at the stem base and slight wilting of the seedling; grade 3, color of necrotic patches at the stem was deep with longitudinal fissure and visible wilting of the seedling; and grade 4, the seedling was dead (modified from Zhou et al., 2010). The disease index was calculated as follows:

$$Dicrease\;index\;(\%)\;=\frac{\sum (\text{Grade}\times \text{Corresponding number of pathogenic seedlings})}{\text{Total number of seedlings investigated }x\text{ Highest disease grade}}x100$$

### Phenotypic Statistics and Experimental Design

Ten days after Foc inoculation, the disease grade of the seedlings was recorded and analyzed. The extremely resistant and extremely sensitive seedlings were separately pooled into an R-pool and S-pool, respectively. Genomic regions that showed signatures of resistance to FW were detected by whole genome resequencing of the DNA from the two parents and the two pools. Regions that underwent specific selection in the opposite directions were selected (Hiroki et al., 2013). The reads in the R- and S-pools were mapped to the Cucumber (Chinese Long) v2 Genome (http://cucurbitgenomics.org/organism/2). The single nucleotide polymorphisms (SNP)-index and Euclidean distance of the two pools were calculated and compared. The Euclidean distance was estimated as described by Hill et al. (2013). The SNP-indexes of the two pools were different because of genotype selection and knock-on effects. QTLs related to the FW resistance trait were roughly located by taking the intersection of the above two results (SNP-index and Euclidean distance).

### Genetic Analysis

The FW resistance in the six generations of cucumber (P_1_, P_2_, F_1_, B_1_, B_2_, and F_2_) was analyzed using the mixed major gene plus polygene inheritance model (Gai and Wang, 1998). The maximum log-likelihood value and Akaike information criterion (AIC) were obtained by estimating the parameters of each generation and component distributions using the iterated EMC (IEMC) algorithm (Gai and Wang, 1998). After repeated iteration, the algorithm converged to a relatively stable and consistent result. The optimal model was selected according to the AIC, and the corresponding component distribution parameters were obtained. The first- and second-order parameters of the optimal model were estimated using a least squares method.

### DNA Extraction, Identification of InDel Markers, and Genotyping

Total genomic DNA was extracted from cotyledons of the parent seedlings using the Cetyltrimethyl Ammonium Bromide (CTAB) -acidic phenol extraction method (Xu et al., 2017). The concentration and quality of the extracted DNA were determined by ultraviolet spectrophotometer (Thermo Fisher, USA) and 1% agarose gel electrophoresis. The primer pairs used for screening for InDel markers were designed using Primer Premier 5.0 with the Cucumber v2 Genome sequence as the reference. For each sample, the PCR mixture (20 µl total volume) contained 2 µl 10× buffer, 1 µl dNTPs (10 mM), 1 µl primer F (50 ng/µl), 1 µl primer R (50 ng/µl), 1 µl DNA, 0.4 µl Taq DNA polymerase (10 U/µl), and 13.6 µl diethyl pyrocarbonate water (DEPC water). The touchdown PCR amplifications were performed using an Eppendorf Mastercycler Pro (Eppendorf). Subsequently, 1 µl PCR product was detected and analyzed by polyacrylamide gel electrophoresis.

PCR and polyacrylamide gel electrophoresis were used to identify polymorphisms in the F_2_ generation using the parent Superina and Rijiecheng plants, and the F_1_ plants are the reference. By combining the genotype and phenotype of the F_2_ seedlings, we determined the location of the interval associated with resistance on the cucumber genome sequence. We designed new InDel marker primers in the resistance interval to fine-map the interval associated with resistance and repeated this until the interval was small or until it contained no new markers.

### Validation of Gene Expression by Real-Time PCR (qPCR)

We have transcriptome data for Rijiecheng, a relatively resistant line, at 0, 24, 48, 96, and 192 h after inoculation with Foc (unpublished data). We used the Foc-inoculated and water-inoculated Rijiecheng seedlings to verify the expression levels of candidate genes by qPCR. Total RNA of each sample was isolated using a Mini BEST Plant RNA Extraction Kit (TaKaRa, China) and then dissolved in Ultra Pure™ DNase/RNase-free distilled water (Invitrogen, USA). The total RNA was reverse-transcribed using a PrimeScript™ RT reagent kit with genomic DNA (gDNA) eraser (TaKaRa, China). Primer sequences were designed using Beacon Designer 7.0 and screened using SeqHunter 1.0 The qPCRs were performed using SYBR^®^ Premix Ex Taq™ (TaKaRa, China), according to the manufacturer’s instructions. SYBR Green PCR cycling was performed on an IQTM5 Multicolor Real-Time PCR Detection System (Bio-Rad, USA) using 20 µl samples as follows: 95 for 3 min, followed by 39 cycles of 95℃ for 10 s, 60 for 20 s, and 72 for 20 s. The relative quantization of gene expression was calculated and normalized to tubulin alpha chain (*Csa4G000580*). Three biological replicates from each condition were used for qPCRs.

## Results

### Variations in FW Phenotypes Among the Cucumber Parents and Segregated Populations

**Figure 1:**
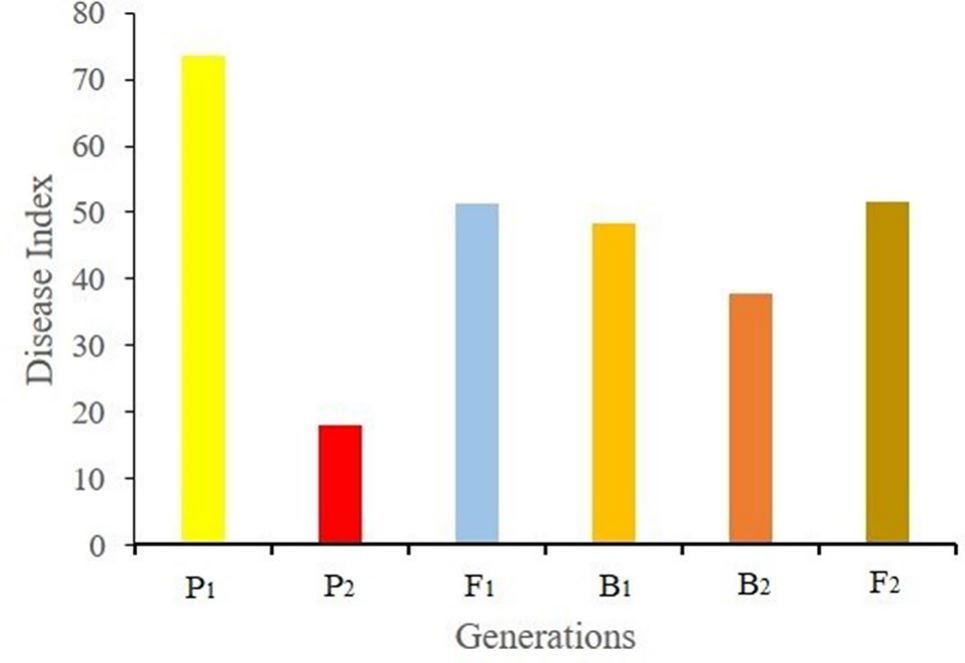
Disease index of each generation. F_2_ plants were obtained from the cross between Superina (female parent, P_1_) and Rijiecheng (male parent, P_2_). Superina (female parent, P_1_) and Rijiecheng (male parent, P_2_) were used to construct different generations, namely F_1_, B_1_ (F_1_× Superina), B_2_ (F_1_× Rijiecheng), and F_2_.

**Figure 2:**
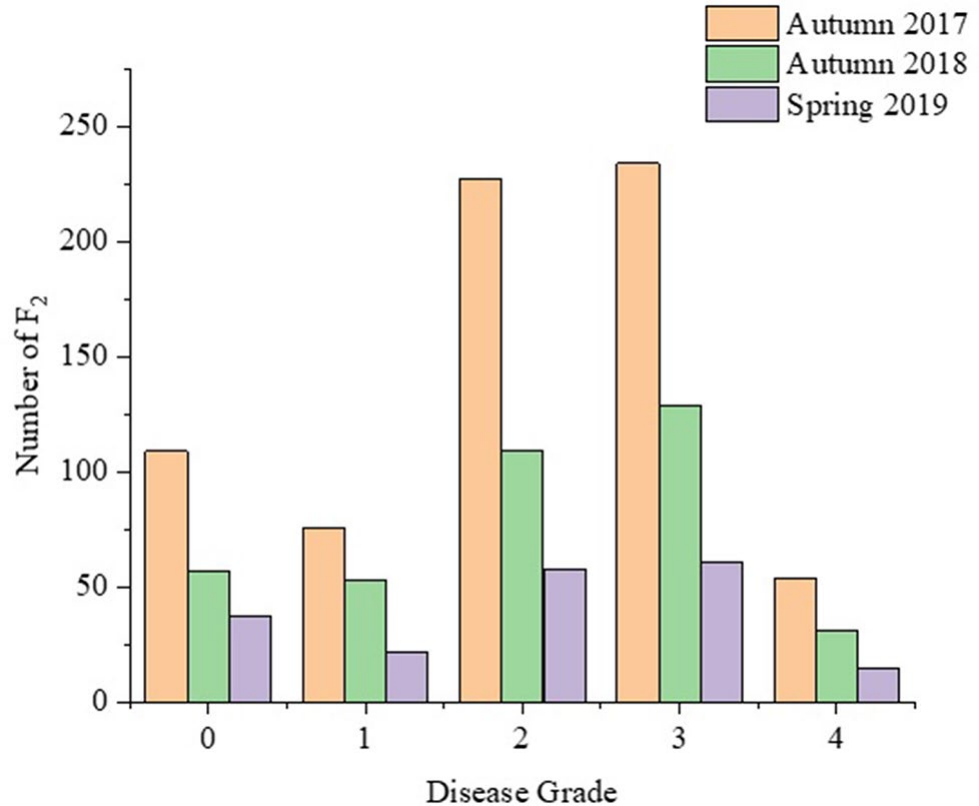
Frequency distribution of the disease grades among the F_2_ population. The disease grade was recorded 10 days after inoculation in Autumn 2017 and 2018, and Spring 2019.

Ten days after Foc inoculation, the disease symptom grades and disease indexes of the seedlings of different generation were recorded and calculated. The disease indexes of P_1_, P_2_, F_1_, B_1_, B_2_, and F_2_ were 73.21, 18.13, 51.43, 48.24, 37.68, and 51.71, respectively (**Figure 1**). The average disease index of F_1_ plants was higher than the mid parent value, indicating that the resistance traits of F_1_ plants tended to be from the male parent Rijiecheng. The disease indexes of the two backcross generations (B_1_ and B_2_) shifted towards the backcross parent. The F_2_ population showed positive correlation between the distribution of disease grades and obvious quantitative genetic characteristics using three replicates (**Figure 2**), which corresponds to the genetic characteristics of a mixed major gene plus polygene inheritance model.

### Best-Fitting Genetic Model for FW Resistance and the Affecting Factors

To obtain a genetic model of cucumber FW resistance, we analyzed the segregation of the resistant phenotype among the six generations using the mixed major gene plus polygene inheritance model. We analyzed and screened a total of 18 genetic models including five categories by combining the maximum log-likelihood value and AIC. The five categories were as follows: one major gene model, two major genes model, polygene model, one major gene plus polygene model, and two major genes plus polygene model (**Table 1**). We selected the two major genes plus polygene models (E, E-1, E-2, and E-3) that had the smallest AIC values as the candidate models. Fitness tests of these four models, including equal distribution (U12, U22, and U32), Smirnov (nW2), and Kolmogorov tests (Dn) (Xu et al., 2017), indicated the significance levels for the E, E-1, E-2, and E-3 models were 7, 1, 11, and 11, respectively (**Table 2**). Finally, we selected the E-1 model (two pairs of additive-dominance-epistatic major genes and additive-dominance polygenes) as the optimal model for resistance to FW on the basis of the combined AIC values and goodness-of-fit test.

**Table 1:** Maximum log-likelihood values (MLVs) and Akaike information criterion (AIC) values under various genetic models estimated using the IECM algorithm.

Model	MLV	AIC	Model	MLV	AIC
A-1	−1,479.132812	2,966.265625	D	−1,466.053711	2,956.107422
A-2	−1,479.893311	2,965.786621	D-3	−1,469.131348	2,954.262695
A-3	−1,482.719971	2,971.439941	D-4	−1,464.250366	2,944.500732
B-1	−1,442.165039	2,904.330078	E	−1,433.539062	2,903.078125*
B-2	−1,455.200073	2,922.400146	E-1	−1,440.745305	2,911.490723*
B-3	−1,585.298706	3,178.597412	E-2	−1,437.410400	2,896.820801*
B-4	−1,484.792725	2,975.585449	E-3	−1,437.569580	2,893.139160*
C	−1,466.125488	2,952.250977	E-4	−1,459.107178	2,934.214355
C-1	−1.469.656250	2,953.312500	E-5	−1,459.093994	2,936.187988

***A***, one major gene model; ***B***, two major genes model; ***C***, polygene model; ***D***, one major gene plus polygene model; ***E***, two major genes plus polygene model. * indicated the four candidate models with smallest AIC values.

**Table 2:** Goodness-of-fit of the two major genes plus polygene models of resistance to FW.

Model	Generation	Maximum log-likelihood estimates of component distribution parameters				
		**U** **_1_** **^2^**	**U** **_2_** **^2^**	**U** **_3_** **^2^**	**_n_** **W** **^2^**	**D** **_n_**
E	P_1_	0.03 (0.86)	0.33 (0.56)	2.73 (0.10) *	0.72 (>0.05)	0.30 (>0.05)
	P_2_	0.12 (0.73)	0.01 (0.92)	2.91 (0.09) *	0.62(>0.05)	0.27 (>0.05)
	F_1_	0.01 (0.94)	0.17 (0.68)	3.80 (0.05) *	0.72 (>0.05)	0.28 (>0.05)
	B_1_	1.96 (0.16) *	1.58 (0.21) *	0.16 (0.69)	0.58 (>0.05)	0.28 (>0.05)
	B_2_	1.23 (0.27) *	1.46 (0.23) *	0.30 (0.59)	0.55 (>0.05)	0.25 (>0.05)
E-1	P_1_	0.09 (0.76)	0.11 (0.74)	0.02 (0.88)	0.60 (>0.05)	0.28 (>0.05)
	P_2_	0.00 (1.00)	0.01 (0.92)	0.18 (0.67)	0.52 (>0.05)	0.24 (>0.05)
	F1	0.00 (1.00)	0.01 (0.91)	0.19 (0.66)	0.58 (>0.05)	0.25 (>0.05)
	B_1_	0.01 (0.92)	0.00 (0.95)	0.45 (0.50)	0.36 (>0.05)	0.19 (>0.05)
	B_2_	0.03 (0.87)	0.04 (0.85)	0.02 (0.89)	0.40 (>0.05)	0.21 (>0.05)
	F_2_	0.00 (0.97)	0.08 (0.78)	1.61 (0.21) *	3.06 (>0.05)	0.16 (>0.05)
E-2	P_1_	0.08 (0.78)	0.02 (0.90)	2.55 (0.11) *	0.71 (>0.05)	0.27 (>0.05)
	P_2_	0.54 (0.46) *	0.07 (0.80)	3.33 (0.07) *	0.67 (>0.05)	0.29 (>0.05)
	F_1_	0.00 (0.98)	0.23 (0.64)	3.98 (0.05) *	0.73 (>0.05)	0.28 (>0.05)
	B_1_	8.40 (0.00) *	9.22 (0.00) *	0.85 (0.36) *	1.25 (>0.05)	0.37 (>0.05)
	B_2_	1.77 (0.18) *	2.28 (0.13) *	0.80 (0.37) *	0.63 (>0.05)	0.26 (>0.05)
	F_2_	0.00 (0.97)	0.20 (0.66)	2.76 (0.10) *	3.15 (>0.05)	0.16 (>0.05)
E-3	P_1_	0.00 (0.97)	0.08 (0.78)	1.61 (0.21) *	3.06 (>0.05)	0.16 (>0.05)
	P_2_	0.08 (0.78)	0.03 (0.87)	3.06 (0.08) *	0.62 (>0.05)	0.26 (>0.05)
	F_1_	0.05 (0.83)	0.52 (0.47) *	4.27 (0.04) *	0.74 (>0.05)	0.30 (>0.05)
	B_1_	7.82 (0.01) *	8.58 (0.00) *	0.79 (0.37) *	1.19 (>0.05)	0.36 (>0.05)
	B_2_	1.45 (0.23) *	1.82 (0.18) *	0.53 (0.47) *	0.60 (>0.05)	0.25 (>0.05)
	F_2_	0.15 (0.70)	0.00 (0.98)	2.43 (0.12) *	3.16 (> 0.05)	0.17 (>0.05)

U_1_^2^, U_2_^2^, and U_3_^2^, uniformity test; _n_W^2^, Smirnov test; D_n_, Kolmogorov test; FW, Fusarium wilt. * indicates significance at 0.05.

To determine the genetic effects of major genes, we estimated first- and second-order distribution parameters for resistance to FW in the F_1_ generation using the optimal E-1 model (**Table 3**). The additive effect values of the major gene and polygene were both 0.05, and the dominant effect values of the major gene and polygene were 0.99 and 0.57, respectively, which indicated that the additive effect of the major gene was consistent with that of the polygene, whereas the dominant effect of the major gene was greater than that of the polygene. This suggested that the contribution rates of major gene and polygene were consistent with inheritance of resistance to FW. The heritability of major gene $\left(h_{mg}^{2}\right)$ from B_1_, B_2_, and F_2_ generations was 22.91%, 29.70%, and 59.73%; the heritability of polygene $\left(h_{pg}^{2}\right)$ from B_1_, B_2_, and F_2_ was 29.92%, 21.78%, and 2.43%; and the environmental variance $\left(\sigma_{e}^{2}\right)$ accounted for 47.62%, 48.52%5, and 37.84% of the phenotypic variance $\left(\sigma_{p}^{2}\right)$ for B_1_, B_2_ and F_2_, respectively. These results indicated that environmental factors had a large effect on cucumber resistance to FW.

**Table 3:** Estimates of genetic parameters for resistance to FW in the F_1_ plants (Superina×Rijecheng) cross under the E-1 model

1st parameter	Estimate	2nd parameter	Estimate		
			B_1_	B_2_	F_2_
*m*	0.99	$\sigma_{p}^{2}$	1.26	1.23	1.58
*da*	0.05	$\sigma_{mg}^{2}$	0.29	0.37	0.94
*ha*	0.99	$\sigma_{pg}^{2}$	0.38	0.27	0.04
*db*	0.05	$\sigma_{e}^{2}$	0.60	0.60	0.60
*hb*	0.57	$h_{mg}^{2}$	22.91	29.70	59.73
*ha*/*da*	20.27	$h_{pg}^{2}$	29.92	21.78	2.43
*hb*/*db*	11.94	${\;}_{1}-h_{mg+pg}^{2}$	47.17	48.52	37.84

### Identification of the Major Effect FW QTL by BSA

**Figure 3:**
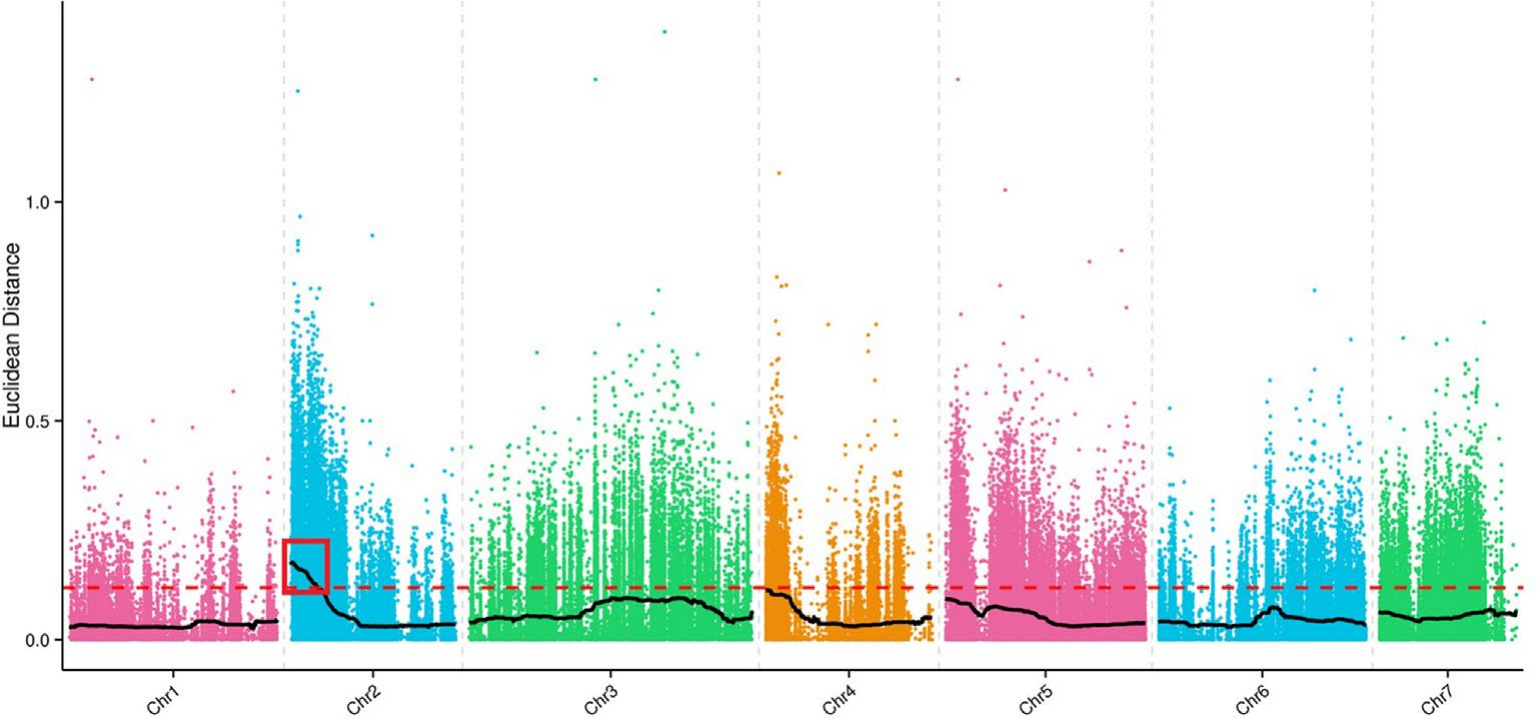
Identification of the major effect *Fusarium* wilt (FW) quantitative trait locus (QTL). Colored points indicate the Euclidean distance values of every SNP on each of the chromosomes. The black lines indicate the Euclidean distance values after matching. The red dotted line is the threshold line. Values above the threshold line were used to select the candidate FW trait-related interval as shown in the red box.

A total of 214,270,191 clean reads from the transcriptomes of Superina, Rijiecheng, S-pool, and R-pool were aligned to the Cucumber v2 Genome sequence; the Q30-level was >95%, and the GC-contents was >35%. The detailed statistics for each transcriptome including numbers of clean reads, numbers of bases, and average depth are given in **Table 4**. We identified a 3.78 Mb region on chromosome 2 (Chr2) that contained 625 genes that had an association threshold (Euclidean distance) of 0.12. One region on Chr2 with a total length of 1.91 Mb and containing 319 genes had an association threshold of 0.25 after fitting the △SNP-index. We used the intersection of these two results to identify the region of the genome associated FW resistance. The identified region was on Chr2, was 1.91 Mb long, and contained 319 genes (**Figure 3**), and was designated as the major effect QTL (*fw2.1*) related to the FW resistance trait.

**Table 4:** Evaluation of sample sequencing data.

Name	Clean Reads	Clean Base	Q30 (%)	GC (%)	Average Depth	Radio of Average Depth (%)
Superina	30,821,023	9,212,830,776	93.13	36.97	21	98.56
Rijiecheng	30,766,127	9,197,436,830	93.15	37.23	23	98.80
S-pool	76,119,559	22,745,447,354	92.91	37.30	58	99.10
R-pool	76,572,482	22,865,387,058	92.90	37.24	60	99.12

### Fine Mapping QTL *fw2.1* to 0.6 Mb on Chr2

**Figure 4:**
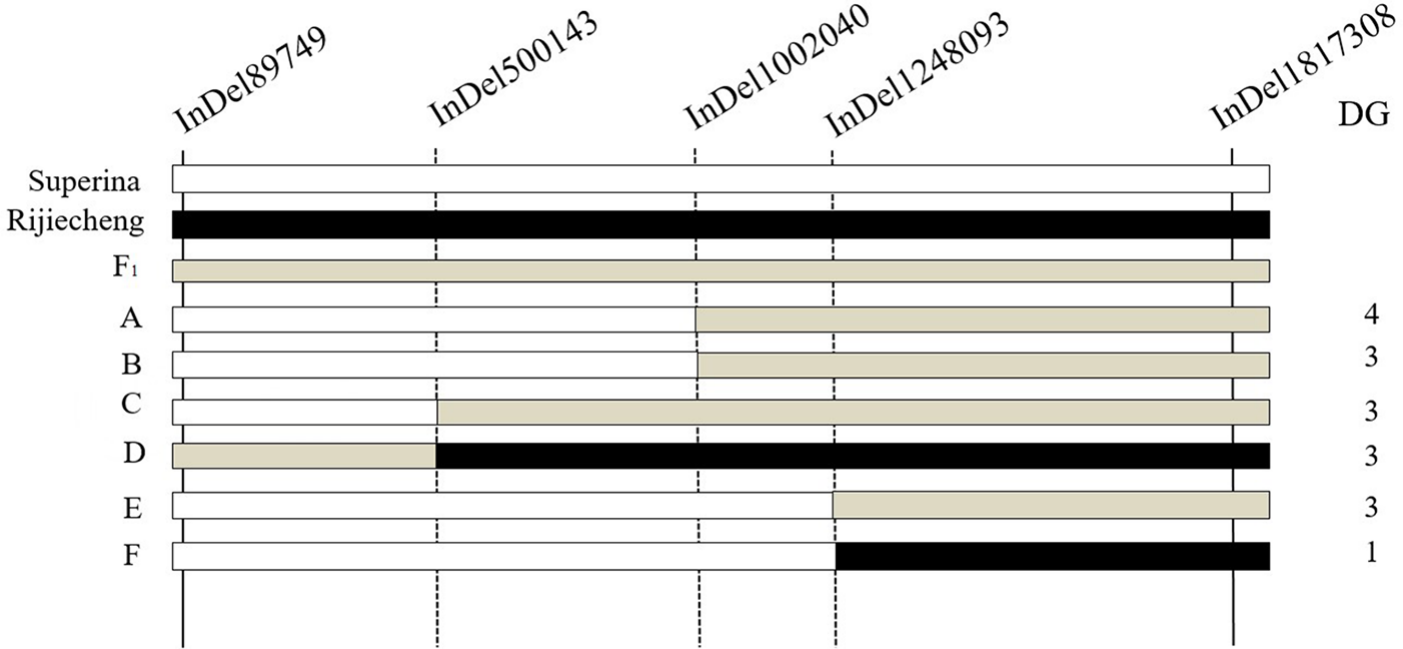
Fine mapping of the major effect QTL *fw2.1* on chromosome 2. The *fw2.1* QTL was located between markers InDel1248093 and InDel1817308, shown in the black box. White bars indicate *Fusarium oxysporum* f. sp. *cucumerinum* (Foc)–sensitive Superina genotype; black bars indicate Foc-resistant Rijiecheng genotype; grey bars indicate F_1_ genotypes. DG is the disease grade. **(A**–**F)** indicate the six different genotypes that were detected among the F_2_ plants.

To narrow down the major effect QTL identified by BSA, whole genome resequencing of the two parents was performed to confirm the high-quality InDels. We genotyped 500 F_2_ plants using two polymorphic InDel markers (InDel89749 and InDel1817308). Three other InDel markers that were evenly distributed in the major effect QTL interval were designed. Six different genotypes were detected among the F_2_ plants; however, two of them had disease grades 0 or 2 (**Figure 4**). By combining the genotypes and phenotypes of the F_2_ seedlings, the candidate interval was located between InDel1248093 and InDel1817308, which are at a physical distance of 569,215 bp in a region that contains 80 genes. Thus, the major effect QTL was fine-mapped to an approximately 0.60 Mb interval (from 1,248,093 to 1,817,308 bp) on Chr2 of the Cucumber v2 Genome assembly.

### Analysis of Candidate Genes by RNA-Seq

**Figure 5:**
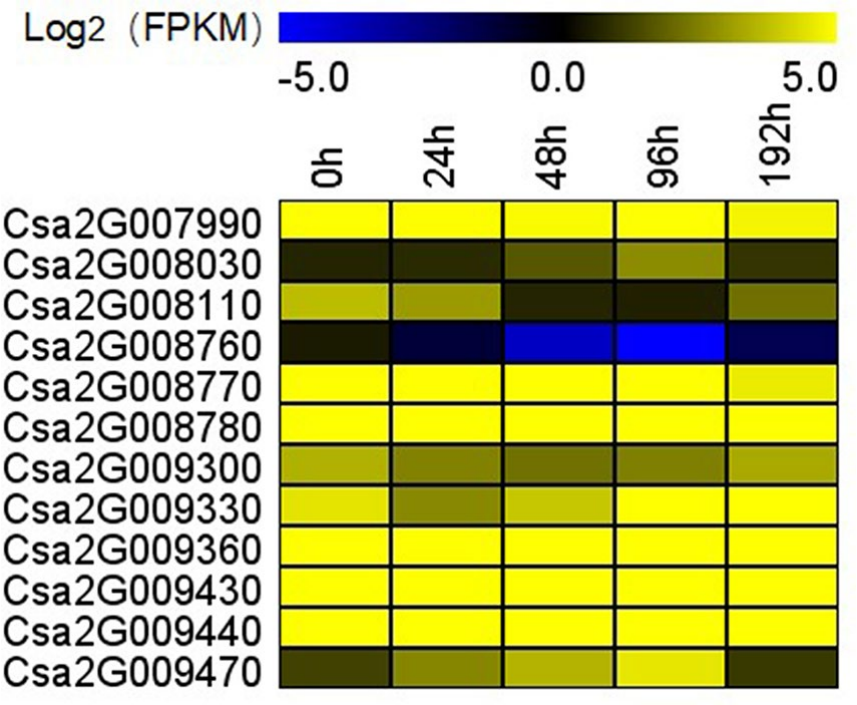
Transcriptional profiles of 12 candidate genes in the major effect QTL *fw2.1* on chromosome 2 at different times after inoculation. The expression data were converted to Log_2_ fragments per kilobase million (FPKM) to measure the expression levels of the FW resistance genes. The color scale indicates fold changes in the expression levels of the genes induced by FW.

**Figure 6:**
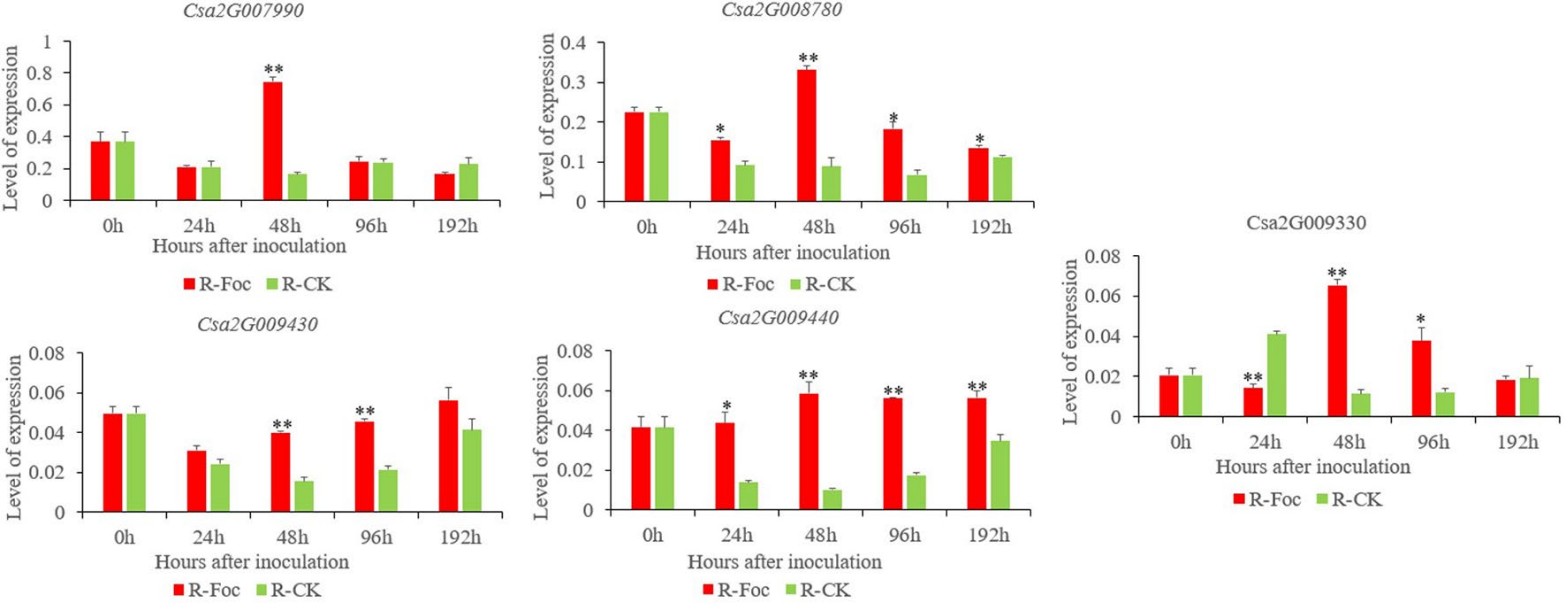
Relative expression patterns of five candidate genes in Superina and Rijiecheng plants inoculated with Foc and water control check (CK) . Each bar represents the average expression level of three independent biological replicates. Error bars show standard errors of the average values. * P ≤ 0.01–0.05 and ** P < 0.01 relative to the expression prior to inoculation with water.

To confirm the FW resistance genes, we analyzed the 80 candidate genes in the major effect QTL using RNA-sequencing (RNA-seq). We combined the RNA-seq data with the previously obtained transcriptome data [national center for biotechnology information’s (NCBI’s) sequence readarchive (SRA) database: PRJNA472169] to detect differentially expressed genes (DEGs) in the 0.60 Mb interval on Chr2. All the DEGs were positively regulated, except for *Csa2G008760*. DEGs were defined as having a false discovery rate ≤0.01 and fold change ≥2 or ≤−2. Twelve genes were found to be differentially induced by FW (**Figure 5**), namely, *Csa2G007990* (calmodulin), *Csa2G008030* [probable Guanosine diphosphate (GDP)-mannose transporter 2], *Csa2G008110* (monogalactosyldiacylglycerol synthase), *Csa2G008760* (chitinase), *Csa2G008770* (adenylate kinase), *Csa2G008780* and *Csa2G009330* (unknown proteins), *Csa2G009300* (DNA replication licensing factor MCM5), *Csa2G009360* (RING-type finger protein 126), *Csa2G009430* (transmembrane protein), *Csa2G009440* (serine-rich protein), and *Csa2G009470* (betaine aldehyde dehydrogenase). We also analyzed the expression patterns of the 12 candidate genes in Rijiecheng by qPCR (**Figure 6**; **Supplementary Figure 1**). The primers of these 12 genes are shown in **Supplementary Table 1**. We found that 5 of the 12 genes were significantly induced by FW, and their relative expression levels were higher in Foc-inoculated Rijiecheng than in the water-inoculated plants.

## Discussion

Several studies on the inheritance of resistance in cucumber investigated quantitative resistance traits controlled by multiple genes (Pierce and Wehner, 1990; Liu et al., 2003; Wang et al., 2005) and qualitative resistance traits controlled by a single gene (Netzer et al., 1977; Vakalounakis 2015). Contradictory results were obtained, possibly because there are different physiological races of Foc with different pathogenicities in different countries (Weng et al., 1989; Armsrong et al., 1987). We investigated the inheritance of cucumber FW resistance using Rijiecheng and Superina as the parents and concluded that the resistance in Rijiecheng was quantitative and the inheritance of FW resistance was controlled by multiple genes. This result was confirmed for F_2_ plants grown in different years and seasons, indicating that resistance to FW was a quantitative trait in cucumber. In dry pea, an F_2_ population segregating for high levels of resistance to *Fusarium solani* f. sp. Pisi also had a disease reaction phenotype in repeated greenhouse trials (Coyne et al., 2019).

Many studies on molecular linkage markers and genetic mapping of cucumber powdery mildew and downy mildew have been reported (Zhang et al., 2011; Zhang et al., 2013a; Zhang et al., 2013b; Xu et al., 2016). However, the genetic mapping of cucumber FW has rarely been reported (Zhang et al., 2014). One simple sequence repeat (SSR) marker linked to cucumber *Foc2.1* was identified in a genetic interval of 5.98 cM (Wang, 2005) and validated among 46 germplasms. The accuracy rate of this SSR marker for selecting resistant germplasm was 87.88%. Zhang et al. (2014) found one major QTL on cucumber Chr2 (SSR03084–SSR17631) for FW resistance in a genetic distance of 2.4 cM. Zhou et al. (2015) concluded that cucumber *Foc4* resistance to FW was located between SSR17631 and SSR00684 on Chr2. In this study, we found five InDel markers with polymorphisms in P_1_, P_2_, and F_1_ at different physical positions on Chr2 by BSA and successfully identified the major effect QTL on Chr2 with a physical distance of 0.60 Mb (InDel1248093–InDel1817308). Notably, the physical positions of all these QTLs are different: 2,526,888–3,262,528 (Zhang et al., 2014), 3,255,681–3,262,528 (Zhou et al., 2015), and 1,248,093–1,817,308 in the present study.

We found 12 DEGs in *fw2.1* by combining BSA with RNA-seq after inoculating the plants with Foc infection and taking samples at different times. In previous studies, RNA-seq data were used to explore key susceptible genes in cucumber that responded to foliage diseases (Zheng et al., 2019). Liu et al. (2019) suggested that the differences in gene expression following cucumber mosaic virus (CMW) infection might explain the different resistance levels of two lines on the basis of RNA-seq data. Shi et al. (2018) analyzed the RNA-seq data of a recombinant inbred line and located a QTL on Chr5 that contained nine genes in cucumber infected with CMW. We found five candidate genes by combining QTL mapping and RNA-seq for resistance to FW in cucumber. *Csa2G007990* encodes calmodulin and is highly expressed in cucumber. Naveed et al. (2019) found that when calmodulin bound to the effector peptide protein Avrblb2, it incited a hypersensitive response. *Csa2G009430* encodes a transmembrane protein that has been widely studied in animal disease resistance, but not in plants. *Csa2G009440* encodes a serine-rich protein, which has been linked to the ability of bacteria to attach to the hosts. For example, Santiago et al. (2007) suggested that serine-rich region may be involved in the negative regulation of phytochrome signal transduction, which is known to yield a hyperactive photoreceptor. *Csa2G008780* and *Csa2G009330* are highly expressed in cucumber, and their expression levels were significantly higher in Foc-inoculated plants than that in water-inoculated plants. The functions of these two genes are still unknown and require further study to determine their characteristics and functions. We plan to verify the functions of the five candidate genes using other methods such as virus-induced gene silencing and overexpression. Our results will help to better understand the genetic mechanisms and provide a strong basis for fine mapping of the major effect QTL and for cloning the candidate genes for resistance to FW in cucumber.

## Data Availability Statement

The transcriptome data associated with this study can be found in NCBI using accession number PRJNA472169 (https://www.ncbi.nlm.nih.gov/bioproject/PRJNA472169).

## Author Contributions

XC and XX conceived the experiment. JD and JX performed the research and wrote the manuscript. JD and QX analyzed the data. All authors reviewed and approved this submission.

## Funding

This research was supported financially by the Jiangsu Agriculture Science and Technology Innovation Fund (CX (17) 2004) and SCX (19) 3029, Special Funds for Three New Agricultural Projects in Jiangsu Province (SXGC [2017] 303), the National Natural Science Foundation of China (31902015), and Project of breeding for the major new agricultural variety of Jiangsu Province (PZCZ201720).

## Conflict of Interest

The authors declare that the research was conducted in the absence of any commercial or financial relationships that could be construed as a potential conflict of interest.

## Supplementary Material

SUPPLEMENTARY FIGURE 1 Relative expression patterns of the seven rejected candidate genes in the Superina and Rijiecheng plants inoculated with Foc and water (CK). Each bar represents the average expression level of three independent biological replicates. Error bars show standard errors of the average values. *P ≤0.01–0.05 and **P <0.01 relative to the expression prior to inoculation by water.
