# Supplementary figures and images for: Inheritance and Quantitative Trait Locus Mapping of Fusarium Wilt Resistance in Cucumber
Source: Front Plant Sci. 2019 Dec 2;10:1425. doi: 10.3389/fpls.2019.01425 (PMC6900741; doi:10.3389/fpls.2019.01425)

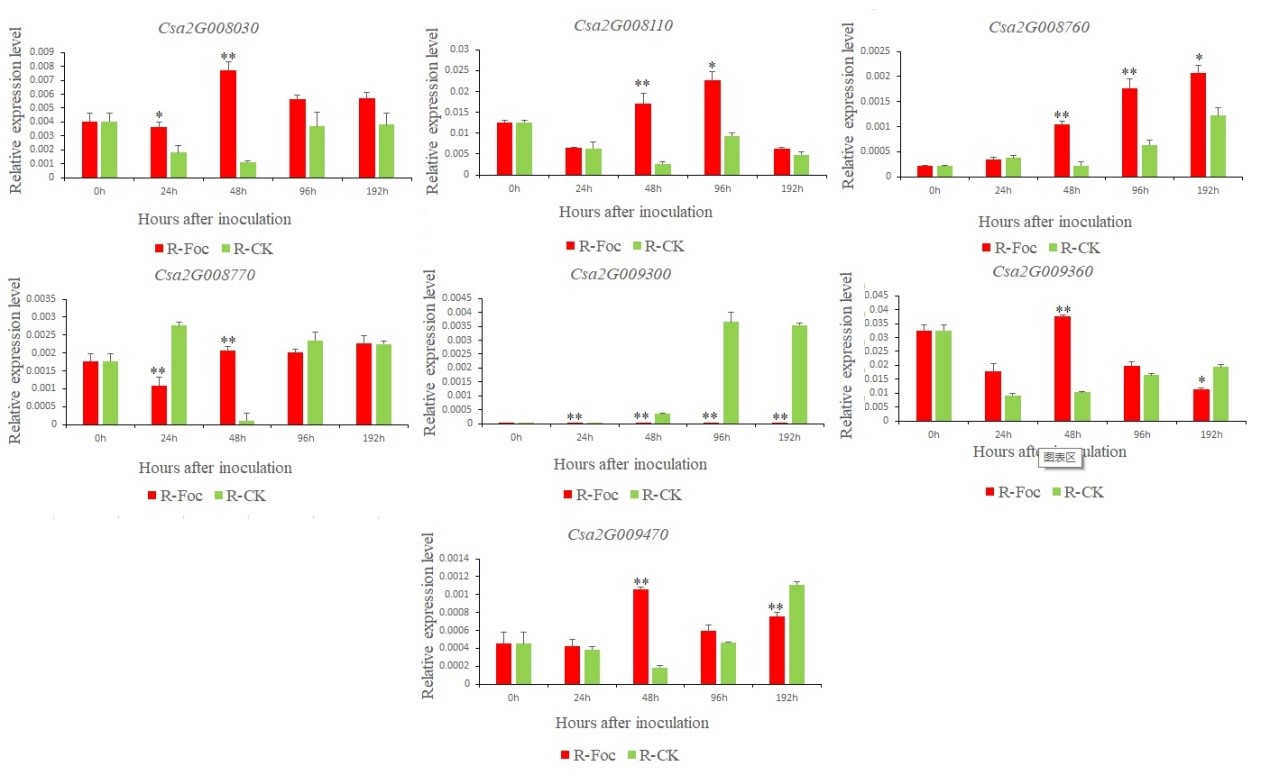

Supplement: SUPPLEMENTARY FIGURE 1 — Relative expression patterns of the seven rejected candidate genes in the Superina and Rijiecheng plants inoculated with Foc and water (CK). Each bar represents the average expression level of three independent biological replicates. Error bars show standard errors of the average values. *P ≤0.01–0.05 and **P <0.01 relative to the expression prior to inoculation by water. [file Image_1.jpeg]
