# Supplementary material for: Inheritance and Quantitative Trait Locus Mapping of Fusarium Wilt Resistance in Cucumber
Source: Front Plant Sci. 2019 Dec 2;10:1425. doi: 10.3389/fpls.2019.01425 (PMC6900741; doi:10.3389/fpls.2019.01425)
Supplement: Supplementary file 2 [file Table_1.docx]

**SUPPLEMENTAL TABLE 1** DGEs located in the major effect QTL interval.

| Gene ID | Gene Annotation | Chinese Long location | Primer |
| --- | --- | --- | --- |
| *Csa2G007990* | Calmodulin | 1347969 .. 1348997 | GAAGATGAGGAAGATGAGT, CTAATGAACACAACACCAA |
| *Csa2G008030* | Probable GDP-mannose transporter 2 | 1384375 .. 1389713 | CAGGGATTTGTCATTTGATACC, CGAGCAATAGAGGCAAGATA |
| *Csa2G008110* | Monogalactosyldiacylglycerol synthase | 1427981 .. 1431664 | ACCATTAGCCTCTACTCTTG, CATCCACTTCTCCATCTGT |
| *Csa2G008760* | Chitinase 2 | 1514322 .. 1516483 | GGTGGTTGTGTTAGTATC, TCTTCTGATTCGTTGATG |
| *Csa2G008770* | Adenylate kinase, putative | 1518250 .. 1526995 | CGGAGAATCTTAGAATACAT, ATCTTCATCGTCAACTTC |
| *Csa2G008780* | Unknown protein | 1526272 .. 1526883 | GTGATGATTCTTGTGGTAA, ATCCTTGAAGTCCGTTAA |
| *Csa2G009300* | DNA replication licensing factor MCM5 | 1565028 .. 1570784 | ACAATGCTACAGAATAAC, CGCTTAGTCTTACAATAG |
| *Csa2G009330* | Unknown protein | 1589836 .. 1590619 | TCTGCTTCCTCTTCACCTT, ATCATCGTCCTCGCTCAA |
| *Csa2G009360* | RING finger protein 126 | 1601375 .. 1604357 | TTCATCATCTTCGTCACAG, CATCCAACCGTTCCTTAG |
| *Csa2G009430* | Transmembrane protein | 1661794 .. 1665443 | TGTCGGTGTATCTGTCTTC, GTGCCAATATCGTCAATCC |
| *Csa2G009440* | Serine-rich protein | 1671114 .. 1672637 | TAGAATTGGAGGCGTTGA, TGCTGATGAGAAGAAGGT |
| *Csa2G009470* | Betaine aldehyde dehydrogenase | 1686076 .. 1690146 | GAAGAAGGTGTTAGGATT, TAGTTGAATGGAGGAATG |
